# Establishment and validation of clinical prediction model for hemorrhoid recurrence after procedure for prolapse and hemorrhoids

**DOI:** 10.1097/MD.0000000000034062

**Published:** 2023-06-30

**Authors:** Yulong Zhang, Shiwei Sun, Zhenguo Han

**Affiliations:** a Third Hospital of Shanxi Medical University, Shanxi Bethune Hospital, Shanxi Academy of Medical Sciences Tongji Shanxi Hospital, Taiyuan, China; b General Surgery Department, Shanxi Bethune Hospital, Taiyuan, China.

**Keywords:** hemorrhoid, procedure for prolapse and hemorrhoids, recurrence, risk factors

## Abstract

This study aimed to establish a clinical model to predict the risk of hemorrhoid recurrence after procedure for prolapse and hemorrhoids. The clinical data of patients who underwent stapler hemorrhoidal mucosal circumcision in Shanxi Bethune Hospital from April 2014 to June 2017 were collected retrospectively and followed up regularly after the operation. Finally, 415 patients were included, which were divided into training group (n = 290) and verification group (n = 125). Logistic regression method was used to select meaningful predictors. The prediction model was constructed with nomographs, and the model was evaluated with correction curve, receiver operating characteristic curve and C index. The decision analysis curve was used to determine the clinical utility of the nomogram. Birth history, muscle attachment, postoperative anal urgency, anal resting pressure, postoperative nutritional index, body mass index, Wexner score, and hemorrhoid grading were included in the nomogram. The area under the curve of the prediction model was 0.813 and 0.679, respectively, in the training and verification groups, and 0.839 and 0.746, respectively, for the 5-year recurrence rate. The C index (0.737) and clinical decision curve showed that the model had high clinical practical value. The prediction model of hemorrhoid recurrence risk after hemorrhoidectomy based on multiple clinical indicators can be used for individualized prediction of hemorrhoid recurrence in patients after hemorrhoidectomy, and early intervention measures can be given to individuals with a high recurrence risk to reduce the risk of recurrence.

## 1. Introduction

After decades of development of hemorrhoid treatment methods, reducing the recurrence of hemorrhoid prolapse has become key to various treatments.^[1]^An anastomotic suprahemorrhoidal mucosal circumferential hemorrhoidectomy (procedure for prolapse and hemorrhoids [PPH]) was described by Longo in 1998.^[2]^ PPH has become an effective option for the treatment of hemorrhoids because of its simplicity, short operative time, minimal pain, and quick recovery.^[3,4]^ Therefore, this procedure has been widely used in many centers. However, in the long term, PPH surgery has a high recurrence rate of prolapse, which varies widely from different literature descriptions, ranging from approximately 4% to 26%.^[4–8]^ Recurrence of hemorrhoids decreases patient satisfaction with treatment, causes inconvenience to patients’ lives, and may also incur the cost of retreatment. In this study, we analyzed the clinical data of patients after PPH to identify the risk factors associated with hemorrhoidal recurrence and constructed a statistical model to predict hemorrhoidal recurrence after PPH.

## 2. Materials and methods

### 2.1. Patient

The clinical data of patients with hemorrhoids who visited Shanxi Baiqiuen Hospital for PPH surgery between April 2014 and June 2017 were retrospectively collected to analyze the risk factors for hemorrhoid recurrence, and the model was established and validated. The inclusion criteria were as follows: Patients with hemorrhoids with a clear preoperative diagnosis of grade II or above; Patients undergoing PPH surgery; and The clinical medical history is complete, and the laboratory and examination results required for this study are complete. The exclusion criteria: refusal to participate; imperfect clinical data; missing or less than 60 months of follow-up; death during the study period; diagnosis of hemorrhoids but no PPH surgery; and combined rectal cancer. A total of 415 patients were included in this study (see Supplementary documents, Supplemental Digital Content, http://links.lww.com/MD/J155, which contains follow-up data for all patients). Informed consent was obtained from the patients or their family members through a standardized telephone interview. This study was conducted in accordance with the Declaration of Helsinki. This study was approved by the Ethics Committee of the Third Hospital of the Shanxi Medical University (YXLL-2017-131).

### 2.2. Experimental method

A random sampling method was used to divide the patients into training and validation groups in a ratio of 7:3. Data from the training group were used for feature screening and model construction, and data from the validation group were used to validate the model.

### 2.3. Observed indicators

The clinical data of patients retrospectively collected from the electronic medical record system and during follow-up visits to patients included hemorrhoid grade, history of surgery, history of mental illness, years of prolapse, history of diabetes, title of surgeon, whether the mucosa was completely circumcised, whether the specimen contained muscle, amount of intraoperative blood loss, postoperative pain, postoperative anal urgency, postoperative anal resting pressure, postoperative nutritional index (PNI = ABL + 5*LYMPH), days of hospitalization, and postoperative constipation score (Wexner score). Patient demographic information was also collected, including age, sex, body mass index (BMI), smoking history, reproductive history, and alcohol consumption history. Hemorrhoids were graded using the Goligher grading system.^[9]^ Postoperative anal urgency was defined as a sensation of urgency to defecate lasting more than 2 hours. Wexner scoring was performed by emailing the patient and asking him/her or by phone. The closing indicator was whether and when the hemorrhoids recurred, and the time was chosen as the time of recurrence or the last follow-up visit.

### 2.4. Follow up

All patients were followed up every 2 months for the first year after surgery, every 4 months for the 2nd to 3rd years, and once a year for 3 years or more. All patients were required to come to our hospital outpatient clinic or to community and above hospitals for a clear diagnosis of hemorrhoids after physical examination by a specialist; this was defined as a recurrence of hemorrhoids.

### 2.5. Statistics

R 4.2.1 (Vienna Foundation for Statistical Computing, Vienna, Austria) and software packages such as “rms” and “glmnet” are used to process data. All continuous variables did not conform to the normal distribution; therefore, they are represented by the median (interquartile interval). The classification variables were expressed as frequencies and percentages (%). The patients were randomly divided into training and verification groups at a ratio of 7:3, and the difference in basic data between each group was compared using the rank sum test or chi-square test. Single factor and multi-factor Cox regression analyses were conducted using the “rms” software package, and the independent influencing factors are obtained through the “glmnet” software package and Lasso regression analysis, and a nomogram model was established. The receiver operating characteristic (ROC) curve and area under the curve (AUC) were used to verify the predictive effectiveness of the model. Verify the nomogram model with the bootstrap resampling method and draw the calibration curve to evaluate the consistency of the model and analyze the net benefit to patients through clinical Decision Curve Analysis. *P* values < .05, it is considered statistically significant.

## 3. Results

### 3.1. General information

A total of 415 patients with a median age of 40.00 [31.00, 50.00] years were included in this study. The overall recurrence rate of hemorrhoids is 25% (105/415). Among them, 292 (70.4%) were male and 123 (29.6%) were female. The median constipation score were 3.00 [0.00, 13.75]. There were 284 cases of grade 2 hemorrhoids (68.4%), 89 of grade 3 hemorrhoids (21.4%), and 42 of grade 4 hemorrhoids (10.1%). The baseline data of the patients are presented in Table 1.

**Table 1:** Baseline characteristics of the patients.

Variables	Total cohort	Recurrence in 5 years		Z/χ^2^	*P* value
	(n = 415)	Yes (n = 105)	No (n = 310)		
Sex					
Male	292 (70.4)	64 (21.9)	228 (78.1)	5.967	.015*
Female	123 (29.6)	41 (33.3)	82 (66.7)		
Age	40.00 [31.00, 50.00]	42.00 [32.00, 51.00]	39.50 [30.00, 50.00]	1.757	.079
Grade					
2	284 (68.4)	59 (20.8)	225 (79.2)	11.715	.003*
3	89 (21.4)	28 (31.5)	61 (68.5)		
4	42 (10.1)	18 (42.9)	24 (57.1)		
Previous surgery					
None	306 (73.7)	66 (21.6)	240 (78.4)	10.850	.093
Gallbladder	12 (2.9)	4 (33.3)	8 (66.7)		
Appendix	21 (5.1)	6 (28.6)	15 (71.4)		
Uterine	32 (7.7)	13 (40.6)	19 (59.4)		
Anus	32 (7.7)	13 (40.6)	19 (59.4)		
Colon	7 (1.7)	2 (28.6)	5 (71.4)		
Others	5 (1.2)	1 (20.0)	4 (80.0)		
Psychiatric history					
No	389 (93.7)	93 (23.9)	296 (76.1)	6.382	.012*
Yes	26 (6.3)	12 (46.2)	14 (53.8)		
Parturition	0.00 [0.00, 1.00]	0.00 [0.00, 2.00]	0.00 [0.00, 0.00]	3.368	<.001*
Duration of illness	3.00 [1.00, 7.00]	3.00 [1.00, 10.00]	2.00 [1.00, 5.00]	1.259	.193
Diabetes mellitus					
No	384 (92.5)	95 (24.7)	289 (75.3)	0.858	.354
Yes	31 (7.5)	10 (32.3)	21 (67.7)		
Alcohol					
No	328 (79.0)	78 (23.8)	250 (76.2)	1.914	.166
Yes	87 (21.0)	27 (31.0)	60 (69.0)		
Surgeon					
Middle	47 (11.3)	11 (23.4)	36 (76.6)	0.166	.920
Vice-senior	196 (47.2)	49 (25.0)	147 (75.0)		
Senior	172 (41.4)	45 (26.2)	127 (73.8)		
OT	40.00 [30.00, 48.50]	40.00 [34.00, 50.00]	40.00 [30.00, 47.00]	2.257	.023*
CSP					
No	67 (16.1)	15 (22.4)	52 (77.6)	0.359	.549
Yes	348 (83.9)	90 (25.9)	258 (74.1)		
Muscle attachments					
No	120 (28.9)	22 (18.3)	98 (81.7)	4.337	.037*
Yes	295 (71.1)	83 (28.1)	212 (71.9)		
PAU					
No	305 (73.5)	68 (22.3)	237 (77.7)	5.502	.019*
Yes	110 (26.5)	37 (33.6)	73 (66.4)		
BL	15.00 [10.00, 26.00]	20.00 [10.00, 30.00]	15.00 [10.00, 25.00]	1.349	.167
RAP	4.80 [3.10, 6.90]	4.40 [2.00, 6.00]	4.90 [3.60, 7.00]	3.005	.003*
Postoperative nutrient index	51.10 [45.60, 55.25]	49.80 [44.60, 52.70]	51.45 [46.20, 55.88]	2.871	.004*
Hospital stays	7.00 [5.00, 10.00]	6.00 [5.00, 8.00]	7.00 [5.00, 10.00]	2.096	.035*
BMI	23.30 [21.20, 25.00]	23.90 [21.80, 25.80]	23.10 [21.10, 24.60]	2.873	.004*
Wexner score	3.00 [0.00, 13.50]	5.00 [2.00, 19.00]	2.00 [0.00, 7.00]	3.286	<.001*

BL = blood loss, BMI = body mass index, CSP = circular stapling procedure, OT = operation time, PAU = postoperative anal urgency, PNI = postoperative nutrient index, RAP = resting anal pressure.

* *P* < .05.

### 3.2. Risk factor analysis

**Figure 1.**
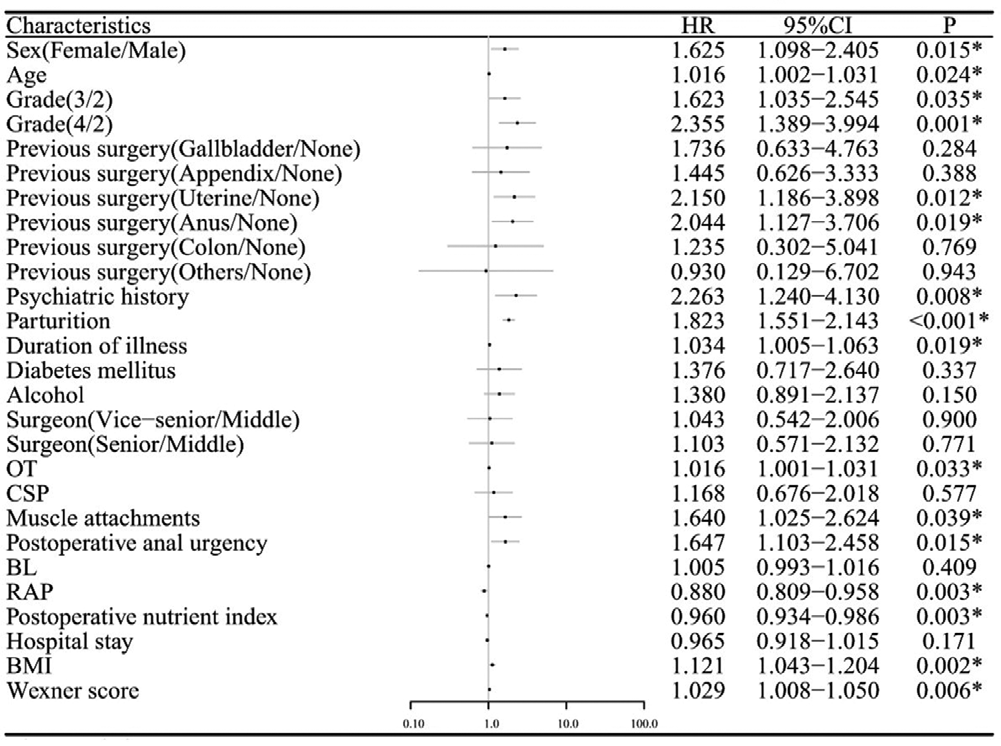
Forest map of risk factors for hemorrhoids recurrence. BL = blood loss, BMI = body mass index, CSP = circular stapling procedure, OT = operation time, RAP = resting anal pressure.

**Figure 2.**
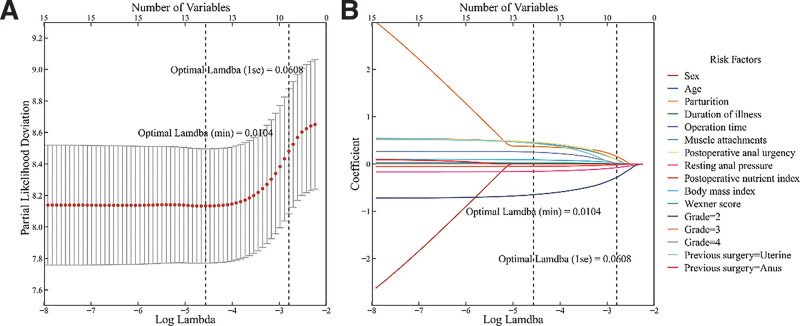
A. 10-fold cross-validation; B. LASSO coefficient curve of 16 variables.

The results of the one-way Cox regression are shown in Figure 1, and the variables with *P* > .05 in the one-way Cox regression were excluded. The remaining variables were converted into multiple dichotomous variables using dummy variables, and the final variable assignments are shown in Table 2. The cases were randomly divided into training and validation groups in a ratio of 7:3. The logarithmic values of the harmonic parameter (λ) were used in the training group. Perform 10-fold cross validation on all variables using LASSO regression, with λ As the logarithmic value changes, the partial likelihood deviation of the vertical coordinate also changes (Fig. 2A). During this process, the coefficient that includes the number of variables is gradually compressed to 0, and the number of variables is also gradually reduced (Fig. 2B). The optimal result corresponds to λ Is 0.0104, corresponding to 1 standard deviation λ Is 0.0608. According to LASSO, 8 influential factors were selected: parturition, muscle attachment, postoperative anal urgency, resting anal pressure, postoperative nutritional index, BMI, Wexner score, hemorrhoid grade II, and hemorrhoid grade IV. (Table 3).

**Table 2:** Variable assignments.

Risk factors	Assignments
Sex	Male = 0, Female = 1
Age	Continuous variable
Parturition	Continuous variable
Duration of illness	Continuous variable
OT	Continuous variable
Muscle attachments	No = 0, Yes = 1
Postoperative anal urgency	No = 0, Yes = 1
RAP	Continuous variable
Postoperative nutrient index	Continuous variable
BMI	Continuous variable
Wexner score	Continuous variable
Grade = II	No = 0, Yes = 1
Grade = III	No = 0, Yes = 1
Grade = IV	No = 0, Yes = 1
Previous surgery = Uterine	No = 0, Yes = 1
Previous surgery = Anus	No = 0, Yes = 1

BMI = body mass index, OT = operation time, RAP = resting anal pressure.

**Table 3:** Risk factors selected by LASSO.

Risk factors	Coefficient	Exp (coef)
Parturition	0.161	1.174
Muscle attachments	0.090	1.094
Postoperative anal urgency	0.080	1.084
RAP	−0.077	0.926
Postoperative nutrient index	−0.017	0.983
BMI	0.024	1.025
Wexner score	0.001	1.001
Grade = 2	−0.285	0.752
Grade = 4	0.0002	1.0002

BMI = body mass index, RAP = resting anal pressure.

### 3.3. Nomograms

**Figure 3.**
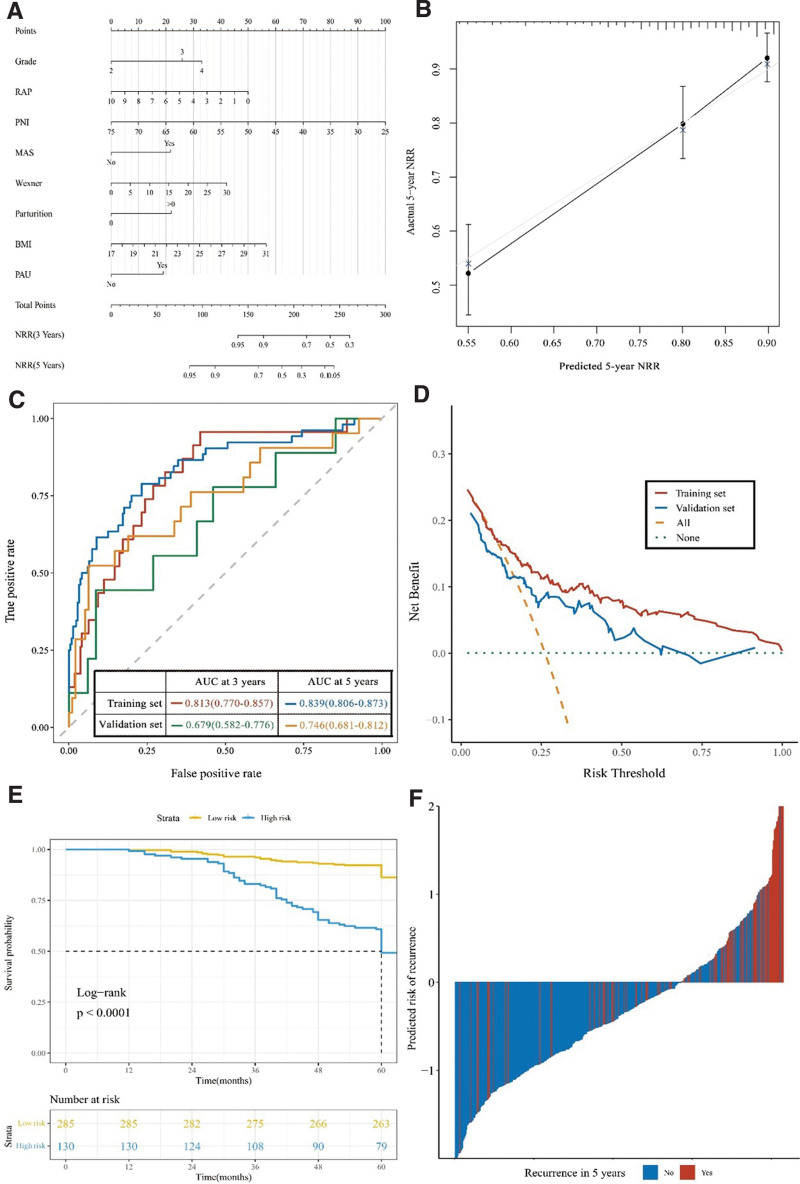
Nomogram of prediction model and its performance. A. nomogram; B. 5 year recurrence rate prediction calibration curve; C. ROC curve in training and verification set; D. Analysis of clinical decision curve of prediction model; E. The Kaplan–Meier curve of risk grouping is predicted according to the model; F. Calculated risk score of each patient in the training and validation set. ROC = receiver operating characteristic.

The LASSO regression model was represented by a column line plot (Fig. 3A). The column line plot was used: the scores corresponding to each predictor of the patient were summed to calculate the total score, and the corresponding risk value identified on the total score line was the probability of non-recurrence of postoperative hemorrhoidal prolapse in that patient.

### 3.4. Validation and performance of nomograms

The C-index of the model was 0.737 (95% CI: 0.689–0.785), and after 1000 resamples of internal validation, the calibration curve fitted well with the ideal curve, indicating that the predicted probability of the model was in good agreement with the actual situation (Fig. 3B). The ROC curves were plotted according to the model fitting results, and the AUCs of the 3-year recurrence rate were 0.813 (95% CI: 0.770–0.857) and 0.679 (95% CI: 0.582–0.776) for the training and validation groups, respectively. The AUCs of the 5-year recurrence rate were 0.839 (95% CI: 0.806–0.873) and 0.746 (95% CI: 0.681–0.812), indicating that the model has a high predictive power (Fig. 3C). By plotting the clinical decision curves based on the model, it was observed that the model could improve the net benefit rate of patients by up to approximately 11% (Fig. 3D). The overall sensitivity of the model was 0.629, the specificity was 0.794, and the Jorden index was 0.423, with high predictive accuracy (Fig. 3E and F). Define the outcome indicator as the outcome at recurrence, with Precision = 0.508, Recall = 0.629, and F1 score = 0.562.

## 4. Discussion

This retrospective study constructed a column line graph to predict hemorrhoid recurrence after PPH based on multiple clinical factors with strong predictive power when the number of predictors was filtered from 16 to 9. Predictors included history of delivery, specimen muscle attachment, postoperative anal urgency, anal resting pressure, postoperative nutritional index, BMI, Wexner score, and grade 2 or 4 hemorrhoids. Multiple indicators can determine the validity of the columnar graph, including the AUC, C-index, ROC curve, and Decision Curve Analysis. To our knowledge, this is the first study to propose a model to predict hemorrhoid recurrence after PPH, using which the risk of hemorrhoid recurrence after PPH can be predicted to promote early clinical intervention and reduce the risk of hemorrhoid recurrence after PPH.

In women with a history of multiple deliveries, the risk of developing hemorrhoids is greatly increased,^[10]^ the process of childbirth strains the connective tissue, leading to a decrease in the tensile strength of the tissue and loss of elasticity. Second, hormonal changes (estrogen, progesterone, etc.) during pregnancy cause a decrease in the collagen content of pelvic tissues.^[11]^ The mechanical stability of the anal cushion, blood vessels, and surrounding tissue anchoring system is reduced,^[12]^ leading to the recurrence of hemorrhoids.^[10]^ A prospective study of 165 pregnant women found that 1/3 of them had persistent external hemorrhoids after delivery.^[13]^ And pregnancy is often complicated by symptoms of constipation,^[14]^ which is a known risk factor for hemorrhoids,^[10,15]^ most of which are accompanied by an uncoordinated defecation process, and this lack of synergy during defecation leads to degenerative disintegration of the connective tissue supporting the anal cushion, resulting in a distal displacement of the anal cushion. A meta-analysis comparing 3 studies found that the prevalence of hemorrhoids was significantly higher in constipated patients than in non-constipated patients.^[16]^ In our study, we investigated the severity of constipation in patients using the Wexner scale and found that the degree of constipation was significantly associated with hemorrhoidal recurrence. Obesity has been shown to be a risk factor for hemorrhoids, and abdominal obesity leads to increased intra-abdominal pressure and perianal venous stasis.^[17]^

The high recurrence rate after grade IV hemorrhoids may be related to incomplete removal of the mucosa, possibly because the volume of prolapsed tissue exceeds the volume of the anastomotic cannula, resulting in insufficient mucosal removal.^[18]^ Zanella et al compared the prognosis of PPH and stapled transanal anastomosis rectal resection and found that stapled transanal anastomosis rectal resection could reduce recurrence by removing more mucosa.^[19,20]^ Some grade II hemorrhoids are suitable for PPH surgery,^[21,22]^ but the number of patients with grade II hemorrhoids included in this study was 297, and the recurrence rate was 21% after 5 years, which is a high recurrence rate and may be related to a less strict grasp of the indications for surgery. Therefore, the indications for the procedure should be strictly controlled, and serious complications and high recurrence rates may occur with the inappropriate use of PPH.^[23,24]^

This study was a retrospective survey, and it was not possible to test the anal resting pressure of patients before surgery and to conduct a comparative study of anal resting pressure before and after surgery, which is a shortcoming of this study. However, our study found that lower than normal anal resting pressure was associated with recurrence of hemorrhoids, and the possible mechanism was a decrease in anal resting pressure and hypertrophy of the anal pad to maintain anal resting pressure, leading to hypertrophic tissue prolapse.^[15]^ Some of the muscle may be accidentally cut when using the anastomosis to remove the mucosa, and this study found that both the presence of muscle in the specimen and a lower than normal postoperative anal resting pressure were risk factors for hemorrhoid recurrence. This suggests that dilatation of the internal sphincter should be avoided as much as possible in future procedures and that relatively gentle retractors can be used to reduce dilatation.

The prognostic nutritional index (PNI) is calculated based on lymphocyte count and serum albumin concentration and usually represents the nutritional and immune status of the patient; PNI is the result of a composite of several factors (nutrition, blood loss, age, diet),^[25]^ and some studies have shown that a low postoperative PNI represents a poor prognosis for patients.^[26]^ There are no studies on the long-term prognosis of PNI after hemorrhoid surgery. However, in our study, we found a correlation between low PNI values and long-term postoperative hemorrhoid recurrence outcomes after surgery by counting PNI values. Our study provides clinical evidence for the use of PNI values in hemorrhoidal disease.

This study had some limitations in that it was a single-center study without external validation of the prediction model, thus limiting the “generalization” ability of the model.

## 5. Conclusion

Clinical predictive models use multifactorial models to estimate the probability of a specific future disease outcome. This study developed and validated a prediction model based on multiple clinical indicators to more accurately predict the risk of hemorrhoid recurrence after PPH, which can help physicians identify patients at higher risk of recurrence after PPH, work with patients to develop treatment plans, enhance collaboration to reduce the inconvenience of life due to recurrence, and reduce the costs incurred for re-treatment after recurrence.

## Author contributions

**Conceptualization:** Zhenguo Han.

**Data curation:** Shiwei Sun.

**Formal analysis:** Yulong Zhang.

**Methodology:** Zhenguo Han.

**Software:** Shiwei Sun.

**Supervision:** Zhenguo Han.

**Validation:** Zhenguo Han.

**Visualization:** Yulong Zhang.

**Writing – original draft:** Yulong Zhang.

## Supplementary Material
